# Airway surface liquid from smokers promotes bacterial growth and biofilm formation via iron-lactoferrin imbalance

**DOI:** 10.1186/s12931-018-0743-x

**Published:** 2018-03-10

**Authors:** Luis G. Vargas Buonfiglio, Jennifer A. Borcherding, Mark Frommelt, Gavin J. Parker, Bryce Duchman, Oriana G. Vanegas Calderón, Ruth Fernandez-Ruiz, Julio E. Noriega, Elizabeth A. Stone, Alicia K. Gerke, Joseph Zabner, Alejandro P. Comellas

**Affiliations:** 1 Department of Internal Medicine, Roy J. and Lucille A. Carver College of Medicine, University of Iowa, 6312 Pappajohn Biomedical Discovery Building. Newton Road, Iowa City, IA 52242 USA; 2 Department of Chemistry, College of Liberal Arts & Sciences, University of Iowa, Iowa City, IA USA; 3 Department of Pediatrics, Roy J. and Lucille A. Carver College of Medicine, University of Iowa, Iowa City, IA USA

## Abstract

**Background:**

Smoking is a leading cause of respiratory infections worldwide. Tobacco particulate matter disrupts iron homeostasis in the lungs and increases the iron content in the airways of smokers. The airway epithelia secrete lactoferrin to quench iron required for bacteria to proliferate and cause lung infections. We hypothesized that smokers would have increased bacterial growth and biofilm formation via iron lactoferrin imbalance.

**Methods:**

We collected bronchoalveolar lavage (BAL) samples from non-smokers and smokers. We challenged these samples using a standard inoculum of *Staphylococcus aureus* and *Pseudomonas aeruginosa* and quantified bacterial growth and biofilm formation. We measured both iron and lactoferrin in the samples. We investigated the effect of supplementing non-smoker BAL with cigarette smoke extract (CSE) or ferric chloride and the effect of supplementing smoker BAL with lactoferrin on bacterial growth and biofilm formation.

**Results:**

BAL from smokers had increased bacterial growth and biofilm formation compared to non-smokers after both *S. aureus* and *P. aeruginosa* challenge. In addition, we found that samples from smokers had a higher iron to lactoferrin ratio. Supplementing the BAL of non-smokers with cigarette smoke extract and ferric chloride increased bacterial growth. Conversely, supplementing the BAL of smokers with lactoferrin had a concentration-dependent decrease in bacterial growth and biofilm formation.

**Conclusion:**

Cigarette smoking produces factors which increase bacterial growth and biofilm formation in the BAL. We propose that smoking disrupts the iron-to-lactoferrin in the airways. This finding offers a new avenue for potential therapeutic interventions to prevent respiratory infections in smokers.

**Electronic supplementary material:**

The online version of this article (10.1186/s12931-018-0743-x) contains supplementary material, which is available to authorized users.

## Background

Respiratory infections are one of the leading causes of mortality worldwide and smoking is considered a risk factor for developing upper and lower respiratory infections [1–3]. In addition, exposure to cigarette smoke is associated with increased risk of airway bacterial colonization compared to non-smokers [4, 5].

The pathogenesis of infectious airway disease caused by cigarette smoke is complex. Smoking damages airway epithelia, increases mucus production, decreases mucociliary clearance, impairs cell immunity and the production of antimicrobial peptides and proteins (AMPs) in the airway [6–11]. Besides impairing the host response to infectious challenges, cigarette smoke can also affect bacterial virulence [12].

The airway surface liquid (ASL) is a layer of fluid covering the airways that is a first line of defense responsible for antimicrobial activity against airborne pathogens. One of the most abundant AMPs present in the ASL is lactoferrin, a bacteriostatic protein that chelates iron, which is required for bacteria to grow and form biofilms [13]. The impairment of AMP activity plays a fundamental role in the origin of infectious lung diseases. Several factors can alter the activity of AMPs such as decreased pH, increased ionic strength especially due to divalent cations such as iron, magnesium, and calcium [14–18].

Tobacco contains particulate matter and multiple chemicals that can potentially alter the iron homeostasis in the lungs [19, 20]. Since iron promotes bacterial growth and biofilm formation and inhibits AMP activity against pathogens, we hypothesized that the ASL from smokers would grow more bacteria and develop more biofilm compared to non-smokers [21–23]. Repeated respiratory infections in smokers influences the development of chronic inflammation and lung function decline leading to chronic obstructive pulmonary disease (COPD) [24, 25].

We have chosen *Staphylococcus aureus* and *Pseudomonas aeruginosa* as models to study relevant culturable airway pathogens. They are both representative airway Gram positive and negative pathogens. *S. aureus* colonizes the nostrils of smokers in a higher prevalence than non-smoking population [26]. This carrier state has been associated with an increased risk of lethal infections by endogenous strains [27]. *Pseudomonas aeruginosa* is a pathogen present in the airways of patients with COPD at both baseline and during exacerbations [28]. This organism is more prevalent in severe COPD and is associated with poor clinical outcomes in hospitalized patients [29, 30]. Therefore, it is relevant to determine mechanisms implicated in increased risk of respiratory infections in smokers.

Sampling ASL is extremely challenging as it is present at a very small volume in the lungs [31]. Therefore, we have used bronchoalveolar lavage as a surrogate of ASL. Bartlett et al. examined the protein composition of bronchoalveolar lavage (BAL) and ASL from new born pigs and found they had 514 protein in common, including AMPs such as lactoferrin, lysozyme and cathelicidins [32]. We challenged the BAL from smokers and non-smokers with bacteria and assessed growth and biofilm formation. We also investigated the effect of supplementing iron, cigarette smoke extract, and lactoferrin to explore the role of iron in the bactericidal and anti-biofilm properties of the airway.

## Methods

### Human BAL collection and processing

We used biobank-stored BAL samples from non-smokers (*n* = 11) and smokers (n = 11) from the study Human Lung Responses to Respiratory Pathogens that aimed to study the relationship of vitamin D levels and the innate defense of the lung against inhaled bacteria. Participants were selected if they were between the age 18–60, if smoker, FEV1 had to be more than 60% of predicted. Participants characteristics were similar between smokers and non-smokers (Table 1). Participants were excluded if they had history of positive tuberculin test or tuberculosis, pneumonia, recent airway infections, antibiotic use or vaccination, were taking vitamins or medications with selected exceptions, were pregnant, breast-feeding, had asthma, diabetes, heart disease or allergy to lidocaine. More detailed inclusion and exclusion criteria for this study were previously published [33]. The collection was approved by the Institutional Review Board at the University of Iowa (IRB# 200607708). BAL collection was performed as previously described [33]. Briefly, after subjects signed an informed consent, and pregnancy was ruled out using a urine test, participants were premedicated with atropine (0.6 mg intramuscularly (IM)), and either morphine (10 mg IM) or meperidine (12.5–25 mg IM). The airways were locally anesthetized using 2–4% lidocaine. A pulmonary physician performed the bronchoscopies by a standard procedure using a flexible bronchoscope (model P160 or P180; Olympus) at the University of Iowa Hospitals and Clinics (Iowa City, Iowa, USA). Under clinical monitoring, five BAL samples (20 mL) were suctioned into a trap container from three segments of the lungs. The liquid in the collection traps was transferred into conical 50 mL centrifuge tubes. Tubes were centrifuged to separate cells from the rest of the BAL. The supernatant of the tubes was pooled into one Falcon® Cell ­Culture Flasks and stored at − 80 °C. The selection of flasks that was retrieved from the biobank were thawed at once on ice, aliquoted into working samples, and stored again at − 80 °C. Working samples were thawed and used once for every experiment to avoid multiple freeze-thaw cycles.

**Table 1:** Comparison of participant characteristics by smoking status

Characteristics	Smokers	Non-Smokers	*p*-value
Age mean	32.8 (11)	37.7 (13)	0.3881
Male gender (%)	54	81	NA
Recovery rate (%)	77.3 (10.5)	70.7 (16.4)	0.2542

Data expressed as mean and standard deviation

### Assessment of BAL bacteriostatic effect on bacteria

To assess bacterial growth, we used bioluminescent *Pseudomonas aeruginosa* Xen 05 and *Staphylococcus aureus* Xen 29 (Caliper Biosciences, USA). It has been reported that Relative Light Units (RLU) correlate closely with Colony Forming Units (CFU) [16]. Briefly, *P. aeruginosa* Xen 05 was cultured overnight in tryptic soy broth (TSB) and then subculture in iron-free media M9 (BD Difco™, USA) overnight at 37 °C, and then washed twice with Phosphate Buffered Saline without calcium and magnesium (PBS−/−). Thereafter, we combined 100 μL of BAL samples with 10 μL of bacteria (~ 5 × 10^5^ CFU) in a 96-well plate (Optiplate-96, Perkin-Elmer, USA). We measured RLU (527 nm) 6 h after bacterial challenge as a surrogate of live bacteria.

*S. aureus* Xen 29 was cultured overnight in TSB. The next day we washed a subculture of mid-log phase bacteria, twice with PBS−/−. Because *S. aureus* does not grow in M9 iron-free media, we resuspended in minimal essential media (10 mM Sodium phosphate buffer, pH 7.4, 100 mM NaCl, 1% TSB). Thereafter, we combined 10 μL of BAL samples with 10 μL of bacteria (~ 5 × 10^5^ CFU) in a Optiplate-96 and measured RLU 30 min after challenge as a surrogate for live bacteria.

To test the effect of lactoferrin supplementation on bacterial growth, we combined 10 μL of increasing concentrations of recombinant lactoferrin from human milk (10, 30, 100, or 300 μg/mL, final concentration) (Sigma-Aldrich) or phosphate buffer control with 90 μL of the BAL for 30 min at 37 °C. Subsequently, we added 10 μL of bacteria (~ 5 × 10^5^ CFU) and measured RLU at 30 min for *S. aureus* and 6 h for *P. aeruginosa* after bacterial challenge.

### Assessment of biofilm formation

We investigated the formation of biofilms in the BAL from smokers and non-smokers using two methods. For *S. aureus* we used a microtiter dish biofilm formation assay as previously described [34]. Briefly, we combined 10 μL of BAL with 190 μL of bacteria Xen 29 (~ 5 × 10^5^ CFU) suspended in minimal media in a 96 well plate. After 48 h, we extracted the liquid, washed the wells with distilled water, stained the biofilm using crystal violet, removed the excess crystal violet with distilled water, dissolved the stain with 30% acetic acid and read the OD_600_ of every well.

To test the effect of lactoferrin supplementation on the smokers BAL biofilm formation, we combined 40 μL of PBS containing varying concentrations of lactoferrin (30, 300, and 1000 μg/mL) or PBS control with 150 μL of BAL samples from smokers and non-smokers and assessed biofilm formation. This mixture was incubated for 30 min at 37 °C. Thereafter, we added 10 μL of Xen 29 *S. aureus* (~ 1.5 × 10^4^ CFUs) to the 96-well plate and incubated for 48 h at 37 °C. Biofilm formation was assessed at 48 h as previously described.

For *P. aeruginosa* we grew PAO1 overnight in TSB. The overnight culture was washed twice with M9 and cultured for 4 h in M9. Subsequently, 50 μL of the bacterial solution (~ 1.5 × 10^7^ CFUs) was added to a 96 well plate containing 100 μL of BAL with a coverslip mounted perpendicular to the bottom of each well. After 48 h, the coverslips were stained using concanavalin-A conjugated with Texas-red. We used confocal microscopy to measure the depth of biofilm formation by quantifying fluorescence. The Z-stacks were used to create surface plots of biofilm growth based on concanavalin A–Texas red intensity. Intensities are expressed as relative fluorescent units (RFUs) as previously described [21]. To test the effect of lactoferrin supplementation on the smokers BAL biofilm formation, we used a final concentration of 300 μg/mL lactoferrin or phosphate buffer control in the samples and assessed biofilm as previously described.

### Cigarette smoke extract (CSE) production

We produced the CSE as previously described with modifications [35]. We filled a 50 mL syringe with 10 mL of media and inserted the filter end of a research cigarette from the University of Kentucky (Code 3R4F) into the wide end of a 1000 μL pipette tip. We lit the cigarette and the narrow end of the pipette was placed into the tip of the 60 mL syringe. The syringe plunger was pulled smoothly to aspirate smoke from the cigarette, through the pipette tip, and into the syringe and mixed with the media by vigorous shaking. We repeated this process of smoke aspiration and media/smoke mixing until one cigarette was consumed. The resulting mixture was filtered through a 0.22 μm filter unit (EMD Millipore). CSE was made fresh for each experiment using the OD_320_ to standardize the solution. An OD_320_ equal to 1.00 corresponded to 100% CSE.

### Bacterial growth in the presence of CSE alone

For *S. aureus,* we combined 50 μL of increasing concentrations of CSE solutions in PBS −/− (0.1, 0.3, 1, or 3%) with 150 μL of ~ 500 CFUs of log phase Xen 29 *S. aureus* bacteria in sodium phosphate buffer (10 mM, 100 mM NaCl, 1% TSB) in a round bottomed 96-well plate for 30 min at 37 °C. After 30 min, each condition was plated onto tryptic soy agar (TSA) plates_,_ incubated overnight at 37 °C, and CFUs were counted.

For *P. aeruginosa,* Xen 05 bacteria were cultured overnight in TSB media and subculture in M9 media for 12 h at 37 °C. We combined 100 μL of bacterial solution in M9 (2.25 × 10^5^ CFUs) with 100 μL of increasing concentration of CSE in M9 (0.01, 0.1, 1, or 3%) in a 96 well-plate and incubated for 18 h at 37 °C. The next day the samples were plated on TSA plates, incubated overnight at 37 °C to count colonies the following day.

### Bacterial growth in BAL supplemented with CSE and ferric chloride

To supplement BAL with CSE we coincubated 50 μL of increasing concentrations of CSE (0.1, 0.3, 1, or 3%) with 140 μL of non-smokers BAL for 30 min. Thereafter, we added 10 μL of *S. aureus* Xen 29 (~ 1.5 × 10^4^ CFU) and measured RLU at 30 min.

For *P. aeruginosa,* we coincubated 100 μL of a combination of non-smokers BAL with either CSE (1%), FeCl_3_ (1 μg/mL) or control with 100 μL of Xen 05 in M9 (~ 1.5 × 10^7^ CFU) in a 96 well-plate. The sealed plate was incubated overnight and RLU were measured at 18 h.

### Measurement of lactoferrin in the BAL

We diluted BAL 1:5 using water. Thereafter, we measured lactoferrin using a human lactoferrin (HLF2) ELISA kit (Abcam, USA) interpolating the unknowns to a standard curve to calculate the lactoferrin concentration in the BAL samples. The data was corrected for the dilution factor.

### Measurement of metals in the BAL

Trace metals in BAL were analyzed using a Thermo X series II inductively coupled plasma mass spectrometer ICP-MS with a collision cell (ThermoFisher Scientific). Samples were spiked with 0.5 mL of a 1 part per million indium solution (Inorganic ventures CGIN1–1) to serve as an internal standard. A 1:10 dilution was performed with 2% nitric acid (Fisher Chemical, Trace metal grade) and final sample volume was 10 mL. Calibration curves were prepared for each analyte of interest from a multi-element standard (Inorganic Ventures QCP-QCS-3 s source). Standards were prepared in concentrations from 1 to 200 parts per billion with the same internal standard. Standards were diluted with 2% nitric acid. Calibration curves were plotted using the response ratio of analyte/internal standard on the Y axis and concentration of analyte on the X axis. The slope was then used to obtain concentration of analyte in samples after a blank subtraction (2% nitric acid). The data was corrected for the dilution factor and samples that were below blank level concentration were considered as below the limit of detection and plotted as 0.

### Data analysis

Data are expressed as mean ± SEM. For BAL growth, we used raw RLU and for the CSE experiments we normalized data as percent of control using this formula:

$$ \mathrm{Percent}\ \mathrm{of}\ \mathrm{live}\ \mathrm{bacteria}=\kern0.5em \frac{\mathrm{RLU}\ \mathrm{from}\ \mathrm{sample}}{\mathrm{RLU}\ \mathrm{from}\ \mathrm{control}\ \mathrm{vehicle}}\ \mathrm{x}\ 100 $$

All experiments had *n* = 11, were done in replicates in at least two independent experiments. The exception was the metal measurements in the BAL that was done once with all samples using the same standard to ensure that they were comparable. We determined the statistical significance between two related groups using paired *t*-tests. We used multiple comparison ANOVA and Kruskal-Wallis to compare three or more concentrations to their respective control. We also used the Pearson test to calculate correlation coefficients. Data analysis was performed using Graph Pad Prism 6.00 (GraphPad Software, California, USA).

## Results

### Bacteria grows more in the BAL from smokers compared to non-smokers

**Fig. 1:**
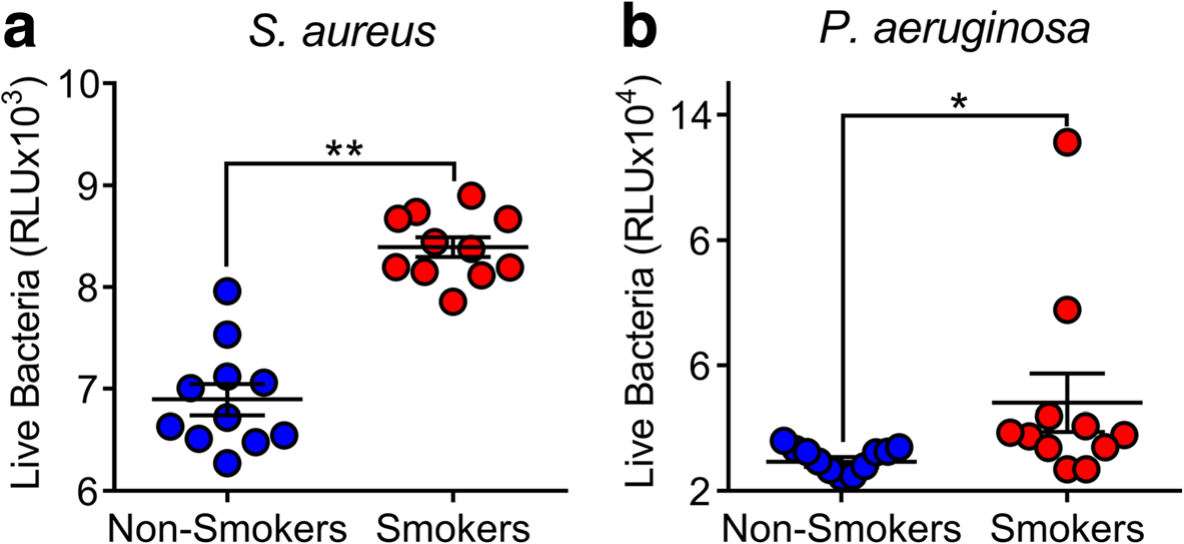
*S. aureus* and *P. aeruginosa* grow more in the BAL from smokers compared to non-smokers. **a** *S. aureus* bacterial growth assessed by RLU at 30 min (**p < 0.0001 by unpaired *t*-test). **b** *P. aeruginosa* bacterial growth by RLU at 6 h. (*p = 0.0493 by unpaired *t*-test)

We hypothesized that BAL from smokers would have increase bacterial growth compared to non-smokers. To investigate this hypothesis we challenged BAL samples, collected via bronchoscopy, with bioluminescent *S. aureus* Xen 29 and measured RLU at 30 min. We found significantly more live bacteria in RLU from the smokers BAL compared to non-smokers. (Fig. 1a). We also challenged the BAL with *Pseudomonas aeruginosa* and found an average of about 64% more RLU at 6 h in the BAL from smokers compared to non-smokers (Fig. 1b). These results suggest that there is increased bacterial growth in the BAL of smokers compared to non-smokers.

### Bacteria develops more biofilm mass in the BAL from smokers compared to non-smokers

**Fig. 2:**
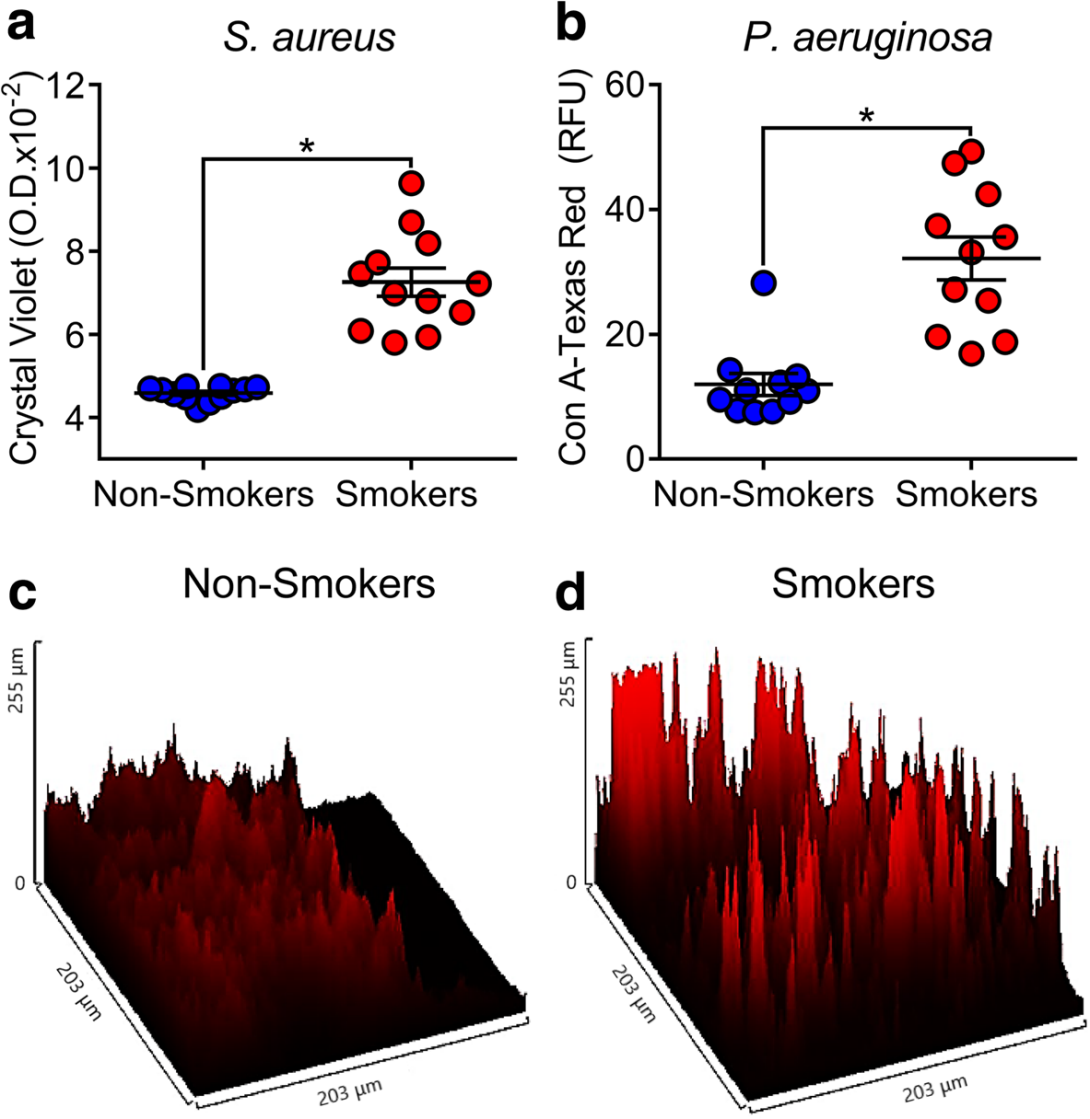
*S. aureus* and *P. aeruginosa* develop more biofilm mass in the BAL from smokers compared to non-smokers. **a** *S. aureus* biofilm formation at 48 h assessed by a microtiter dish biofilm formation assay (*p < 0.0001, by unpaired *t*-test). **b** *P. aeruginosa* biofilm formation at 48 h assessed by fluorescence intensity of Texas-red conjugated to concanavalin-A (Con A-Texas Red) in relative fluorescent units (RFU) (*p < 0.0001, by unpaired *t*-test). **c** Representative surface intensity plot of PAO1 biofilm formation from non-smokers BAL. **d** Representative surface intensity plot of PAO1 biofilm formation from smokers BAL

Since BAL from smokers had more *S. aureus* and *P. aeruginosa* growth compared to non-smokers*,* we investigated the ability of these bacteria to form biofilms. We challenged BAL samples with *S. aureus* and assessed biofilm formation at 48 h by measuring the optical density of crystal violet staining of the biofilm. We found that *S. aureus* in the BAL from smokers had about 52% more biofilm mass compared to non-smokers (Fig. 2a). We also challenged BAL samples with *P. aeruginosa* and quantified the biofilm formation at 48 h by measuring the RFUs of concanavalin-A conjugated with Texas red staining of the bacterial biofilm matrix. We found that *P. aeruginosa* in the BAL of smokers had three times more biofilm matrix compared to non-smokers (Fig. 2b and c). These results suggest that bacteria grow more as a biofilm in the BAL from smokers compared to non-smokers.

### Metals concentration in the BAL

**Fig. 3:**
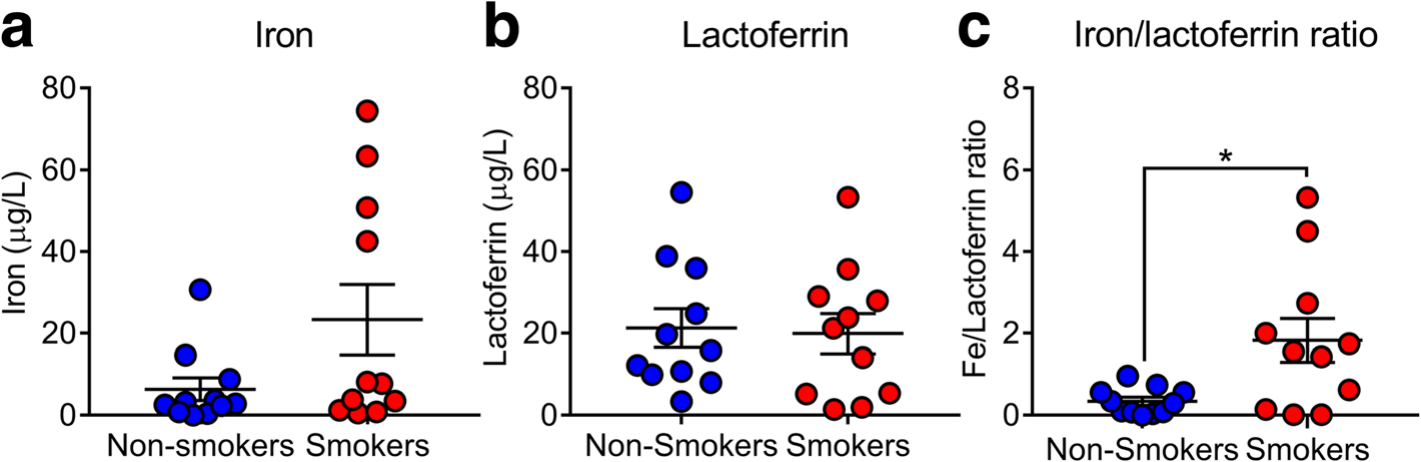
Smokers have increased iron/lactoferrin ratio. **a** Lactoferrin concentration in the BAL from non-smokers and smokers. **b** Iron concentration in the BAL from non-smokers and smokers. **c** Ratio of iron to lactoferrin in the BAL from non-smokers and smokers (*p = 0.0128, by unpaired *t*-test)

Since tobacco contains iron containing particulate matter, we hypothesized that smokers would have higher concentration of metals in the BAL. To investigate this hypothesis, we measured the concentration of selected metals in the BAL of smokers and non-smokers using ICP-MS. We found that some metals, such as aluminum, lead, and vanadium were only detected in the BAL of smokers. Despite that the mean concentration of metals was higher in smokers (e.g. iron was almost four times higher) they were not statistically different (Table 2 and Fig. 3a).

**Table 2:** Concentration of metals present in the BAL from smokers and non-smokers

Element	Metal concentration (ng/mL)		*P*-value^a^
	Non-Smokers BAL	Smokers BAL	
Aluminum	BBL	0.73 (1.89)	NA
Arsenic	3.46 (0.86)	4.24 (1.19)	0.0933
Calcium	811.7 (181.61)	856.8 (183.34)	0.5691
Chromium	0.003 (0.01)	0.03 (0.06)	0.1142
Copper	4.77 (4.9)	4 (2.26)	0.6411
Iron	6.38 (9.12)	23.37 (28.47)	0.0741
Lead	BBL	0.07 (0.16)	NA
Magnesium	389.06 (66.93)	428.76 (110.42)	0.3200
Manganese	0.12 (0.16)	0.21 (0.24)	0.2785
Nickel	0.22 (0.12)	0.5 (0.55)	0.1205
Vanadium	BBL	0.06 (0.02)	NA
Zinc	40.45 (20.99)	48.16 (35.06)	0.5385

Data are expressed in mean (SD). BBL below blank level

^a^Compared by unpaired *t*-test. NA not apply

### Lactoferrin concentration in the BAL

Exposure to cigarette smoke has been associated with increased lactoferrin concentration in human secretions [36, 37]. Therefore, we decided to measure this AMP in our samples. We found that there was not a significant difference in the concentration of lactoferrin in the BAL of smokers compared to non-smokers (Fig. 3b). Because the BALs can have different dilutions, we calculated the ratio of iron to lactoferrin in the BAL samples. We found that the ratio iron/lactoferrin was five times higher in the smoking group compared to non-smokers (Fig. 3c). These data suggest that smokers have an excess of iron in relation to lactoferrin compared to non-smokers.

### Bacteria grow more in non-smokers BAL supplemented with CSE compared to non-supplemented

**Fig. 4:**
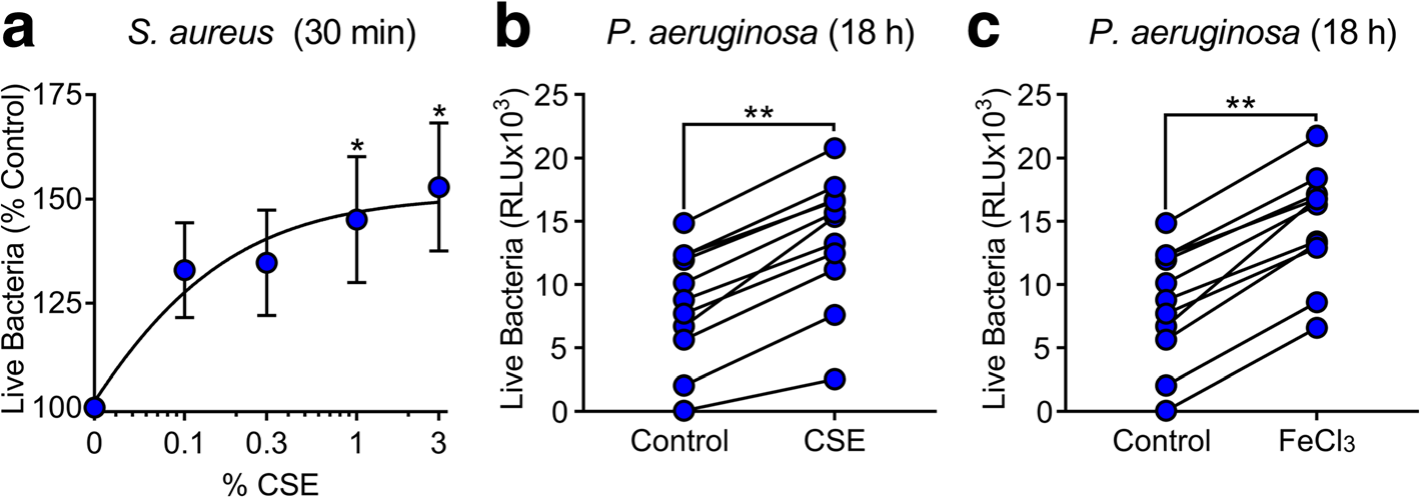
Non-smokers BAL supplemented with CSE has increased bacterial growth. **a** Effect of coincubating BAL from non-smokers with increasing doses of CSE on *S. aureus* bacterial growth (*p < 0.05, compared to control by ANOVA). **b** Effect of coincubating BAL from non-smokers with CSE (1%) on *P. aeruginosa* bacterial growth (**p < 0.0001, by paired *t*-test). **c** Effect of coincubating BAL from non-smokers with FeCl_3_ (1 μg/mL) on *P. aeruginosa* bacterial growth (**p < 0.0001, by paired *t*-test)

Since we found that smokers had an increased iron to lactoferrin ratio in their BAL, we investigated if adding CSE to non-smokers BAL would have a similar effect as smoking on bacterial growth. First, we investigated if CSE alone had an effect on bacterial growth. We combined increasing concentrations of CSE (0.1–3%), with *S. aureus* and *P. aeruginosa* and measured bacterial growth by standard CFU counting. We found that there was no significant increase in bacterial growth in the presence of CSE alone at these concentrations (Additional file 1: Figure S1). These results suggest that CSE by itself is not sufficient to cause increased bacterial growth. We then supplemented BAL samples from non-smokers with CSE at several concentrations and observed a concentration-dependent increase in *S. aureus* growth at 30 min compared to samples exposed to buffer control (Fig. 4a). Thereafter, we exposed BAL from non-smokers to either CSE (0.1%), ferric chloride (1 μg/mL), or control, challenged with *P. aeruginosa*, and read RLU overnight. We found that BAL from non-smokers supplemented with CSE similar to smoking increased bacterial growth of *P. aeruginosa* (Fig. 4b). We found a similar response to CSE when we added ferric chloride to the BAL of non-smokers (Fig. 4c). These results suggest that CSE, similar to iron, enhances bacterial growth.

### BAL from smokers supplemented with lactoferrin reverts bacterial growth and biofilm formation

**Fig. 5:**
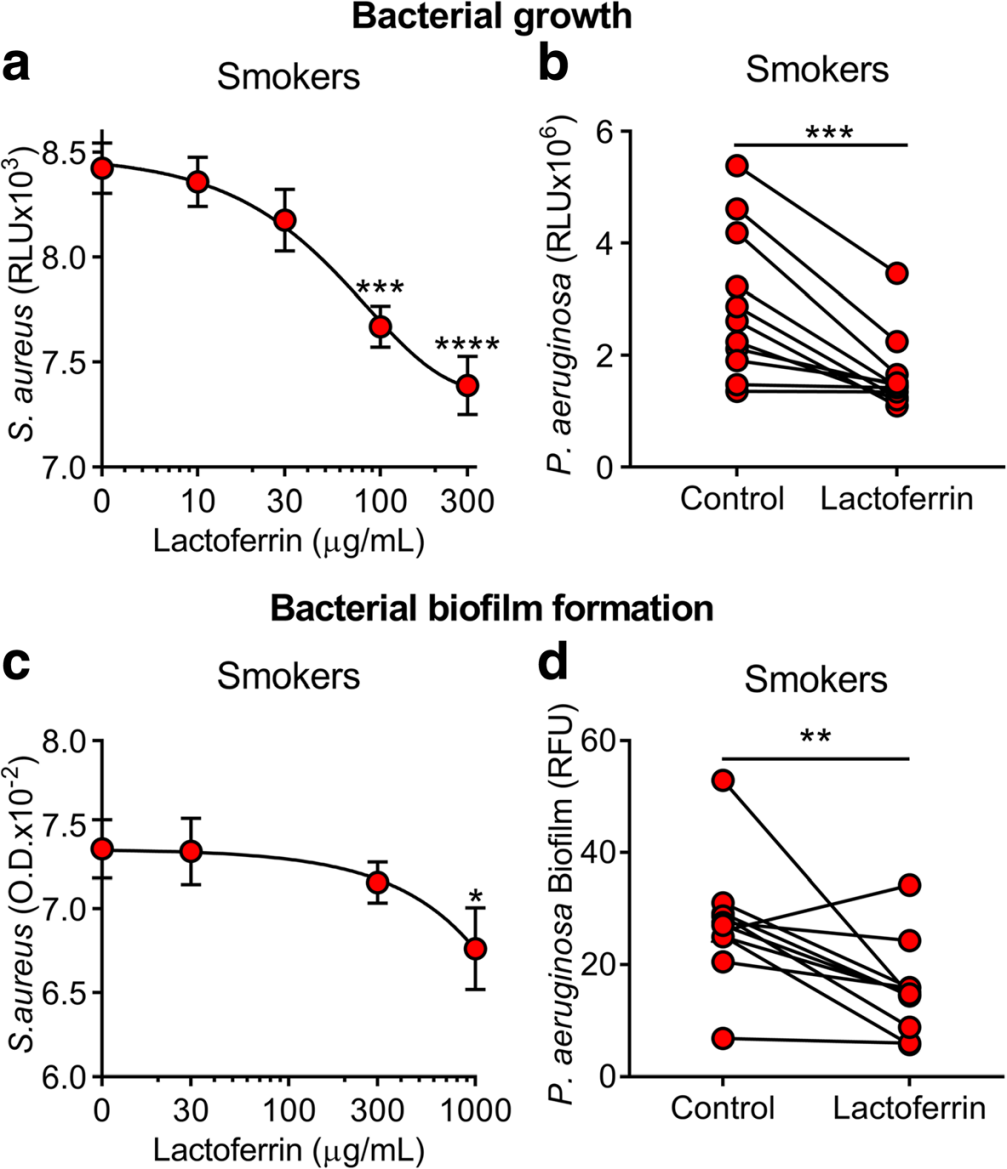
Lactoferrin supplementation decreases bacterial growth and biofilm development in the human BAL from smokers. **a** Effect of coincubating smokers BAL with increasing concentrations of lactoferrin on *S. aureus* growth at 30 min. (***p = 0.0003 and ****p < 0.0001, compared to control by ANOVA). **b** Effect of coincubating BAL from smokers with lactoferrin (300 μg/mL) or control on *P. aeruginosa* growth at 6 h. (***p = 0.0002, by paired *t*-test). **c** Effect of supplementing increasing concentrations of lactoferrin with smokers BAL on *S. aureus* biofilm formation at 48 h. (*p = 0.0188, compared to control by ANOVA). **d** Effect of coincubating BAL from non-smokers with lactoferrin (300 μg/mL) or control on *P. aeruginosa* biofilm formation at 48 h. (**p = 0.0092, by paired *t*-test)

We hypothesized that chelating iron using lactoferrin would prevent the increase in bacterial growth and biofilm formation found in the smokers BAL. To investigate this hypothesis, we coincubated the BAL from smokers with increasing concentration of lactoferrin (10–300 μg/mL), challenged with *S. aureus* (~ 5 × 10^5^ CFU), and measured live bacteria in RLU after 30 min. We found a concentration dependent decrease in live bacteria in the samples from smokers treated with lactoferrin (Fig. 5a). BAL samples from smokers treated with lactoferrin (300 μg/mL) were not statistically different from the non-smokers control (7389 ± 139.5, *n* = 8, vs 6963 ± 202.4, n = 8, respectively *p* = 0.1055 by unpaired *t*-test). We also challenged the BAL samples from smokers treated with lactoferrin (300 μg/mL) with *P. aeruginosa*, and measured RLU at 6 h. We found decreased RLU in the smokers BAL samples treated with lactoferrin compared to control (Fig. 5b). We also supplemented the BAL of smokers with lactoferrin and found a concentration-dependent decrease in the biomass produced by *S. aureus* (Fig. 5c). BAL samples from smokers treated with lactoferrin (1000 μg/mL) were not statistically different from the non-smokers control (0.06763 ± 0.002432, *n* = 11 vs. 0.06835 ± 0.001963, *n* = 11, *p* = 0.8184 by unpaired *t*-test). We also found that supplementing BAL from smokers with lactoferrin (300 μg/mL) significantly decreased the biofilm formation by *P. aeruginosa* at 48 h (Fig. 5d). These results suggest that adding lactoferrin to the smokers BAL decreased bacterial growth and biofilm formation compared to the untreated BAL.

## Discussion

We investigated the bacteriostatic properties of human BAL collected in vivo from smokers and non-smokers using a standard bacterial inoculum. We found that *S. aureus* and *P. aeruginosa* grow more and develop more biofilm in the BAL samples taken from the lungs of smokers compared to non-smokers.

It has been proposed that the surface of the airways is iron-depleted to limit bacterial growth and virulence [38, 39]. Although the study was underpowered to detect significant differences in iron concentrations, we found that smokers had four times higher mean iron concentration in the BAL than non-smokers. This finding is consistent with several reports that have found that smokers have increased iron concentration in their lungs [20, 40–43]. This result and the report of other investigators is in part explained by the disruption of iron homeostasis mechanisms in the lungs of smokers.

Cigarette smoke particles are rich in iron and can directly increase iron concentration in the airways [20] where particulate matter is deposited. Some of the particles are endocytosed and metal oxides on their surface can adsorb intracellular free iron to form ferruginous bodies decreasing its concentration available for the cell. In turn, low iron can be sensed by iron-regulatory proteins that activate iron-responsive elements to post-transcriptionally increase transferrin receptors in the cell basolateral membranes, upregulating iron uptake and further increase the iron content in the lungs [44–46]. In addition, cigarette smoke contains polyhydroxybenzenes that can react with ferritin to release iron [47]. Furthermore, iron in the airways might come from damage in the airway epithelial cells, which results in serum leakage [40].

Increased iron concentration in the airways correlates with the severity of lung disease in cystic fibrosis and chronic bronchitis [38, 48]. It has been proposed that changes in iron homeostasis can affect the susceptibility of the airway to develop infections [46]. Most bacteria rely on a continuous supply of host iron to proliferate [49] and high levels of serum iron can increase the risk of developing active infections such as tuberculosis [50, 51]. In addition, iron nanoparticles can directly impair airway innate mechanism such as AMP activity [21].

We found that other metals such as aluminum, lead, and vanadium that were not present in the airways of non-smokers. Accumulation of metals other than iron has also been observed in the lung and serum of patients with COPD [52, 53]. Some these metal have been showed to impair mechanism involved in airway immunity associated with the pathogenesis of COPD such as decreased release of the AMP and decreased cystic fibrosis transmembrane conductance regulator (CFTR) function [52, 54–56].

When we exposed *S. aureus* and *P. aeruginosa* to only CSE, there was no increased growth compared to a solution control. It has been reported that CSE has variable effects on bacterial growth. In general, it has an inhibitory effect that is greater in Gram positive than Gram negative bacteria [12, 57]. However, these experiments with only CSE were done using doses that would also cause cell death in airway epithelial cells and do not necessarily recapitulate what occurs in the airways [35].

When we supplemented BAL from non-smoking subjects with CSE, as a way to recreate in part the airway microenvironment, we found that CSE impaired BAL bacteriostatic properties. We found a similar impairment of the BAL when we supplemented with iron chloride. These results might suggest that iron bioavailability could be a mechanism for regulating airway antimicrobial activity. One key AMP in the airways is lactoferrin. One of its major function is iron chelation, which reduces the amount of bioavailable iron in biological fluids, including ASL [13]. The function of lactoferrin is affected when saturated with iron [58]. Therefore, we speculate that cigarette smoke contains iron nanoparticles that might not only be a source of iron for bacterial growth but could also inhibit the bacteriostatic properties of the ASL. Conversely, longer times were needed to notice a difference between control BAL and CSE or iron supplemented BAL in *P. aeruginosa* (6 vs 18 h). These results suggest that other factors present in the BAL from smokers such as heme-iron, inflammatory mediators, different composition of AMP might also contribute to the increased bacterial growth.

Both active and passive smoking has been associated with an increase in lactoferrin concentrations in human secretions [36, 37]. In our samples, we found no significant differences in the concentration of lactoferrin between smokers and non-smokers. However, we found an increased ratio of iron to lactoferrin. We speculate that the relative variability in iron/lactoferrin ratio of our samples is responsible to the heterogeneity of some of the results. Especially those experiments using *Pseudomonas* (Figs. 1b, 5b & d). (Fig. 3c). *Pseudomonas* has evolved to efficiently uptake iron by robust and redundant mechanisms [59, 60]. This feature has allowed them to survive in a wide array of environments, such as water currents, plants, nematodes, insects, and in mammals, including humans [61]. Previous studies have suggested that iron in the lungs might be important for *Pseudomonas* airway colonization [62]. As airway disease progresses in COPD, iron deposits in the airways also increases [63]. It is also known that lung diseases such as severe COPD has higher rates of *Pseudomonas* airway colonization. We also speculate that iron/lactoferrin imbalance will also increase the probabilities of being colonized by this pathogen associated with poor clinical outcomes in hospitalized patients [29, 30].

When we supplemented BAL samples with excess lactoferrin we reverted the impaired bacteriostatic activity and biofilm formation observed in the BAL of smokers. One likely mechanism for excess lactoferrin reverting bacterial growth and biofilm formation in smokers is by decreasing bioavailability of iron. Despite that lactoferrin has other antimicrobial mechanisms such as direct binding to LPS, osmotic effect, and bacterial membrane permeabilization, these are also impaired when iron is bound to lactoferrin [64–66]. We acknowledge that the concentration of lactoferrin added to the samples could be considered supraphysiologic. However, commercially available lactoferrin is partially saturated with iron. For this reason, higher doses of AMP were used to observe an effect, particularly in the biofilm experiments.

As a limitation of our study, we acknowledge that *Haemophylus influenzae*, an important organism in COPD exacerbations was not considered for this study. However, this organism needs hemin, an iron containing protoporphyrin and nicotinamide adenine dinucleotide to grow in vitro, the addition of these elements could confound the hypothesis of smoking as a source of iron for bacteria.

Several investigators have recently reported that current and former smokers with preserved lung function by spirometry have increased respiratory symptoms and evidence of airway disease by imaging [67, 68]. This study demonstrates that the imbalance between iron content and lactoferrin abundance in the airways can result in conditions that will impair airway innate immune mechanisms, resulting in an increased risk of respiratory infections. Since the development of airway infections has been proposed as an important mechanism for lung function decline and development of chronic bronchitis, our results provide a potential mechanism for some of the recent reports of respiratory symptoms in smokers without spirometry evidence of COPD [24, 25].

The implications of iron/lactoferrin imbalance in the development of COPD might go beyond increasing bacterial growth. A recent discovery demonstrated that a gene that encodes for an iron receptor protein was associated with dysfunctional mitochondrial iron loading affecting mucociliary clearance and contributing to the development of COPD [69, 70]. In the same study, the use of deferiprone, an USDA approved drug that functions as an iron chelator in a mouse model of COPD, improved features of airway disease progression and acute lung injury such as weight loss, pulmonary inflammation, and decreased mucociliary clearance despite continuous cigarette smoke exposure. Further studies will have to determine the feasibility of this intervention but suggest a promising avenue to prevent the progression from smoking to COPD by iron chelation.

## Conclusion

We conclude that in vivo BAL collected from smokers grows more bacteria and develops more biofilm compared to non-smokers. We presume that excess iron compared to lactoferrin in the airways of smokers impairs the ability of the lungs to control bacterial airway pathogens.

## Additional file

Additional file 1: **Figure S1.** Cigarette smoke extract alone does not increase bacterial growth. (**A**) *S. aureus* growth overnight in the presence of increasing concentrations of CSE assessed by CFU. (**B**) *P. aeruginosa* overnight growth in the presence of increasing concentrations of CSE assessed by CFU. (TIFF 601 kb)

## References

[1] Nuorti JP, Butler JC, Farley MM, Harrison LH, McGeer A, Kolczak MS, Breiman RF (2000). Cigarette smoking and invasive pneumococcal disease. Active bacterial Core surveillance team. N Engl J Med.

[2] Blake GH, Abell TD, Stanley WG (1988). Cigarette smoking and upper respiratory infection among recruits in basic combat training. Ann Intern Med.

[3] Pastor P, Medley F, Murphy TV (1998). Invasive pneumococcal disease in Dallas County, Texas: results from population-based surveillance in 1995. Clin Infect Dis.

[4] Bogaert D, van Belkum A, Sluijter M, Luijendijk A, de Groot R, Rümke HC, Verbrugh HA, Hermans PWM (2004). Colonisation by Streptococcus pneumoniae and Staphylococcus aureus in healthy children. Lancet.

[5] Brook I, Gober AE (2005). Recovery of potential pathogens and interfering bacteria in the nasopharynx of smokers and nonsmokers. Chest.

[6] Bagaitkar J, Demuth DR, Scott DA (2008). Tobacco use increases susceptibility to bacterial infection. Tob Induc Dis.

[7] Arcavi L, Benowitz NL (2004). Cigarette smoking and infection. Arch Intern Med.

[8] Jukosky J, Gosselin BJ, Foley L, Dechen T, Fiering S, Crane-Godreau MA (2015). In vivo cigarette smoke exposure decreases CCL20, SLPI, and BD-1 secretion by human primary nasal epithelial cells. Front Psychiatry.

[9] Qian YJ, Wang X, Gao YF, Duan N, Huang XF, Sun FF, Han XD, Wang WM (2015). Cigarette smoke modulates NOD1 signal pathway and human beta Defensins expression in human oral mucosa. Cell Physiol Biochem.

[10] Crane-Godreau MA, Maccani MA, Eszterhas SK, Warner SL, Jukosky JA, Fiering S (2009). Exposure to cigarette smoke disrupts CCL20-mediated antimicrobial activity in respiratory epithelial cells. Open Immunol J.

[11] Vargas Buonfiglio LG, Cano M, Pezzulo AA, Vanegas Calderon OG, Zabner J, Gerke AK, Comellas AP. Effect of vitamin D_3_ on the antimicrobial activity of human airway surface liquid: preliminary results of a randomised placebo-controlled double-blind trial. BMJ Open Respir Res. 2017;4 10.1136/bmjresp-2017-000211 PMC5531307 28883932

[12] McEachern EK, Hwang JH, Sladewski KM, Nicatia S, Dewitz C, Mathew DP, Nizet V, Crotty Alexander LE (2015). Analysis of the effects of cigarette smoke on staphylococcal virulence phenotypes. Infect Immun.

[13] Singh PK, Parsek MR, Greenberg EP, Welsh MJ (2002). A component of innate immunity prevents bacterial biofilm development. Nature.

[14] Maisetta G, Di Luca M, Esin S, Florio W, Brancatisano FL, Bottai D, Campa M, Batoni G (2008). Evaluation of the inhibitory effects of human serum components on bactericidal activity of human beta defensin 3. Peptides.

[15] Krishnakumari V, Packiyanathan KK, Nagaraj R (2013). Human-beta-defensins-1-3 and analogs do not require proton motive force for antibacterial activity against Escherichia coli. FEMS Microbiol Lett.

[16] Travis SM, Conway BA, Zabner J, Smith JJ, Anderson NN, Singh PK, Greenberg EP, Welsh MJ (1999). Activity of abundant antimicrobials of the human airway. Am J Respir Cell Mol Biol.

[17] Pezzulo AA, Tang XX, Hoegger MJ, Alaiwa MH, Ramachandran S, Moninger TO, Karp PH, Wohlford-Lenane CL, Haagsman HP, van Eijk M (2012). Reduced airway surface pH impairs bacterial killing in the porcine cystic fibrosis lung. Nature.

[18] Abou Alaiwa MH, Reznikov LR, Gansemer ND, Sheets KA, Horswill AR, Stoltz DA, Zabner J, Welsh MJ (2014). pH modulates the activity and synergism of the airway surface liquid antimicrobials beta-defensin-3 and LL-37. Proc Natl Acad Sci U S A.

[19] Musharraf SG, Shoaib M, Siddiqui AJ, Najam-Ul-Haq M, Ahmed A (2012). Quantitative analysis of some important metals and metalloids in tobacco products by inductively coupled plasma-mass spectrometry (ICP-MS). Chem Cent J.

[20] Ghio AJ, Hilborn ED, Stonehuerner JG, Dailey LA, Carter JD, Richards JH, Crissman KM, Foronjy RF, Uyeminami DL, Pinkerton KE (2008). Particulate matter in cigarette smoke alters iron homeostasis to produce a biological effect. Am J Respir Crit Care Med.

[21] Borcherding J, Baltrusaitis J, Chen H, Stebounova L, Wu CM, Rubasinghege G, Mudunkotuwa IA, Caraballo JC, Zabner J, Grassian VH, Comellas AP (2014). Iron oxide nanoparticles induce growth, induce biofilm formation, and inhibit antimicrobial peptide function. Environ Sci Nano.

[22] Borcherding JA, Chen H, Caraballo JC, Baltrusaitis J, Pezzulo AA, Zabner J, Grassian VH, Comellas AP (2013). Coal fly ash impairs airway antimicrobial peptides and increases bacterial growth. PLoS One.

[23] Tunney MM, Einarsson GG, Wei L, Drain M, Klem ER, Cardwell C, Ennis M, Boucher RC, Wolfgang MC, Elborn JS (2013). Lung microbiota and bacterial abundance in patients with bronchiectasis when clinically stable and during exacerbation. Am J Respir Crit Care Med.

[24] Hansbro PM, Starkey MR, Mattes J, Horvat JC (2014). Pulmonary immunity during respiratory infections in early life and the development of severe asthma. Ann Am Thorac Soc.

[25] Hayden LP, Hobbs BD, Cohen RT, Wise RA, Checkley W, Crapo JD, Hersh CP (2015). Childhood pneumonia increases risk for chronic obstructive pulmonary disease: the COPDGene study. Respir Res.

[26] Durmaz R, Tekerekoglu MS, Kalcioglu T, Ozturan O (2001). Nasal carriage of methicillin-resistant Staphylococcus aureus among smokers and cigarette factory workers. New Microbiol.

[27] Wertheim HF, Vos MC, Ott A, van Belkum A, Voss A, Kluytmans JA, van Keulen PH, Vandenbroucke-Grauls CM, Meester MH, Verbrugh HA (2004). Risk and outcome of nosocomial Staphylococcus aureus bacteraemia in nasal carriers versus non-carriers. Lancet.

[28] Murphy TF, Brauer AL, Eschberger K, Lobbins P, Grove L, Cai X, Sethi S (2008). Pseudomonas aeruginosa in chronic obstructive pulmonary disease. Am J Respir Crit Care Med.

[29] Miravitlles M, Espinosa C, Fernandez-Laso E, Martos JA, Maldonado JA, Gallego M (1999). Relationship between bacterial flora in sputum and functional impairment in patients with acute exacerbations of COPD. Study Group of Bacterial Infection in COPD. Chest.

[30] Lin SH, Kuo PH, Hsueh PR, Yang PC, Kuo SH (2007). Sputum bacteriology in hospitalized patients with acute exacerbation of chronic obstructive pulmonary disease in Taiwan with an emphasis on Klebsiella pneumoniae and Pseudomonas aeruginosa. Respirology.

[31] Yager D, Cloutier T, Feldman H, Bastacky J, Drazen JM, Kamm RD (1994). Airway surface liquid thickness as a function of lung volume in small airways of the Guinea pig. J Appl Physiol (1985).

[32] Bartlett JA, Albertolle ME, Wohlford-Lenane C, Pezzulo AA, Zabner J, Niles RK, Fisher SJ, PB MC, Williams KE (2013). Protein composition of bronchoalveolar lavage fluid and airway surface liquid from newborn pigs. Am J Physiol Lung Cell Mol Physiol.

[33] Gerke AK, Pezzulo AA, Tang F, Cavanaugh JE, Bair TB, Phillips E, Powers LS, Monick MM (2014). Effects of vitamin D supplementation on alveolar macrophage gene expression: preliminary results of a randomized, controlled trial. Multidiscip Respir Med.

[34] O'Toole GA. Microtiter dish biofilm formation assay. J Vis Exp. 2011;2437 10.3791/2437 PMC3182663 21307833

[35] Brekman A, Walters MS, Tilley AE, Crystal RG (2014). FOXJ1 prevents cilia growth inhibition by cigarette smoke in human airway epithelium in vitro. Am J Respir Cell Mol Biol.

[36] Thompson AB, Bohling T, Payvandi F, Rennard SI (1990). Lower respiratory tract lactoferrin and lysozyme arise primarily in the airways and are elevated in association with chronic bronchitis. J Lab Clin Med.

[37] Jenabian N, Pouramir M, Motallebnejad M, Bamdadian J, Rahimi-Rad M (2015). Evaluation of the effect of passive smoking on Lactoferrin and AST on 12 - 15 years old children and adolescents. Iran J Pediatr.

[38] Reid DW, Anderson GJ, Lamont IL (2009). Role of lung iron in determining the bacterial and host struggle in cystic fibrosis. Am J Physiol Lung Cell Mol Physiol.

[39] Schaible UE, Kaufmann SH (2004). Iron and microbial infection. Nat Rev Microbiol.

[40] Nelson ME, O'Brien-Ladner AR, Wesselius LJ (1996). Regional variation in iron and iron-binding proteins within the lungs of smokers. Am J Respir Crit Care Med.

[41] McGowan SE, Henley SA (1988). Iron and ferritin contents and distribution in human alveolar macrophages. J Lab Clin Med.

[42] Corhay JL, Weber G, Bury T, Mariz S, Roelandts I, Radermecker MF (1992). Iron content in human alveolar macrophages. Eur Respir J.

[43] Thompson AB, Bohling T, Heires A, Linder J, Rennard SI (1991). Lower respiratory tract iron burden is increased in association with cigarette smoking. J Lab Clin Med.

[44] Ghio AJ, Soukup JM, Dailey LA (1860). Air pollution particles and iron homeostasis. Biochim Biophys Acta.

[45] Khiroya H, Turner AM (2015). The role of iron in pulmonary pathology. Multidiscip Respir Med.

[46] Ghio AJ (2014). Particle exposures and infections. Infection.

[47] Moreno JJ, Foroozesh M, Church DF, Pryor WA (1992). Release of iron from ferritin by aqueous extracts of cigarette smoke. Chem Res Toxicol.

[48] Hunter RC, Asfour F, Dingemans J, Osuna BL, Samad T, Malfroot A, Cornelis P, Newman DK (2013). Ferrous iron is a significant component of bioavailable iron in cystic fibrosis airways. MBio..

[49] Weinberg ED (2009). Iron availability and infection. Biochim Biophys Acta.

[50] Gangaidzo IT, Moyo VM, Mvundura E, Aggrey G, Murphree NL, Khumalo H, Saungweme T, Kasvosve I, Gomo ZA, Rouault T (2001). Association of pulmonary tuberculosis with increased dietary iron. J Infect Dis.

[51] Gordeuk VR, McLaren CE, MacPhail AP, Deichsel G, Bothwell TH (1996). Associations of iron overload in Africa with hepatocellular carcinoma and tuberculosis: Strachan's 1929 thesis revisited. Blood.

[52] Hassan F, Xu X, Nuovo G, Killilea DW, Tyrrell J, Da Tan C, Tarran R, Diaz P, Jee J, Knoell D (2014). Accumulation of metals in GOLD4 COPD lungs is associated with decreased CFTR levels. Respir Res.

[53] Leem AY, Kim SK, Chang J, Kang YA, Kim YS, Park MS, Kim SY, Kim EY, Chung KS, Jung JY (2015). Relationship between blood levels of heavy metals and lung function based on the Korean National Health and nutrition examination survey IV–V. Int J Chron Obstruct Pulmon Dis.

[54] Rab A, Rowe SM, Raju SV, Bebok Z, Matalon S, Collawn JF (2013). Cigarette smoke and CFTR: implications in the pathogenesis of COPD. Am J Physiol Lung Cell Mol Physiol.

[55] Pace E, Ferraro M, Minervini MI, Vitulo P, Pipitone L, Chiappara G, Siena L, Montalbano AM, Johnson M, Gjomarkaj M (2012). Beta defensin-2 is reduced in central but not in distal airways of smoker COPD patients. PLoS One.

[56] Nemery B (1990). Metal toxicity and the respiratory tract. Eur Respir J.

[57] Ertel A, Eng R, Smith SM (1991). THe differential effect of cigarette smoke on the growth of bacteria found in humans. Chest.

[58] Byrd TF, Horwitz MA (1991). Lactoferrin inhibits or promotes legionella pneumophila intracellular multiplication in nonactivated and interferon gamma-activated human monocytes depending upon its degree of iron saturation. Iron-lactoferrin and nonphysiologic iron chelates reverse monocyte activation against legionella pneumophila. J Clin Invest.

[59] Konings AF, Martin LW, Sharples KJ, Roddam LF, Latham R, Reid DW, Lamont IL (2013). Pseudomonas aeruginosa uses multiple pathways to acquire iron during chronic infection in cystic fibrosis lungs. Infect Immun.

[60] Minandri F, Imperi F, Frangipani E, Bonchi C, Visaggio D, Facchini M, Pasquali P, Bragonzi A, Visca P (2016). Role of Iron uptake systems in Pseudomonas aeruginosa virulence and airway infection. Infect Immun.

[61] Cornelis P, Dingemans J (2013). Pseudomonas aeruginosa adapts its iron uptake strategies in function of the type of infections. Front Cell Infect Microbiol.

[62] Reid DW, Carroll V, O'May C, Champion A, Kirov SM (2007). Increased airway iron as a potential factor in the persistence of Pseudomonas aeruginosa infection in cystic fibrosis. Eur Respir J.

[63] Philippot Q, Deslee G, Adair-Kirk TL, Woods JC, Byers D, Conradi S, Dury S, Perotin JM, Lebargy F, Cassan C (2014). Increased iron sequestration in alveolar macrophages in chronic obstructive pulmonary disease. PLoS One.

[64] Ellison RT, Giehl TJ, LaForce FM (1988). Damage of the outer membrane of enteric gram-negative bacteria by lactoferrin and transferrin. Infect Immun.

[65] Ellison RT, Giehl TJ (1991). Killing of gram-negative bacteria by lactoferrin and lysozyme. J Clin Invest.

[66] Appelmelk BJ, An YQ, Geerts M, Thijs BG, de Boer HA, MacLaren DM, de Graaff J, Nuijens JH (1994). Lactoferrin is a lipid A-binding protein. Infect Immun.

[67] Woodruff PG, Barr RG, Bleecker E, Christenson SA, Couper D, Curtis JL, Gouskova NA, Hansel NN, Hoffman EA, Kanner RE (2016). Clinical significance of symptoms in smokers with preserved pulmonary function. N Engl J Med.

[68] Bowler RP, Kim V, Regan E, Williams AAA, Santorico SA, Make BJ, Lynch DA, Hokanson JE, Washko GR, Bercz P (2014). Prediction of acute respiratory disease in current and former smokers with and without COPD. Chest.

[69] Cloonan SM, Glass K, Laucho-Contreras ME, Bhashyam AR, Cervo M, Pabon MA, Konrad C, Polverino F, Siempos II, Perez E (2016). Mitochondrial iron chelation ameliorates cigarette smoke-induced bronchitis and emphysema in mice. Nat Med.

[70] DeMeo DL, Mariani T, Bhattacharya S, Srisuma S, Lange C, Litonjua A, Bueno R, Pillai SG, Lomas DA, Sparrow D (2009). Integration of genomic and genetic approaches implicates IREB2 as a COPD susceptibility gene. Am J Hum Genet.

