# Supplementary figures and images for: Airway surface liquid from smokers promotes bacterial growth and biofilm formation via iron-lactoferrin imbalance
Source: Respir Res. 2018 Mar 10;19:42. doi: 10.1186/s12931-018-0743-x (PMC5845328; doi:10.1186/s12931-018-0743-x)

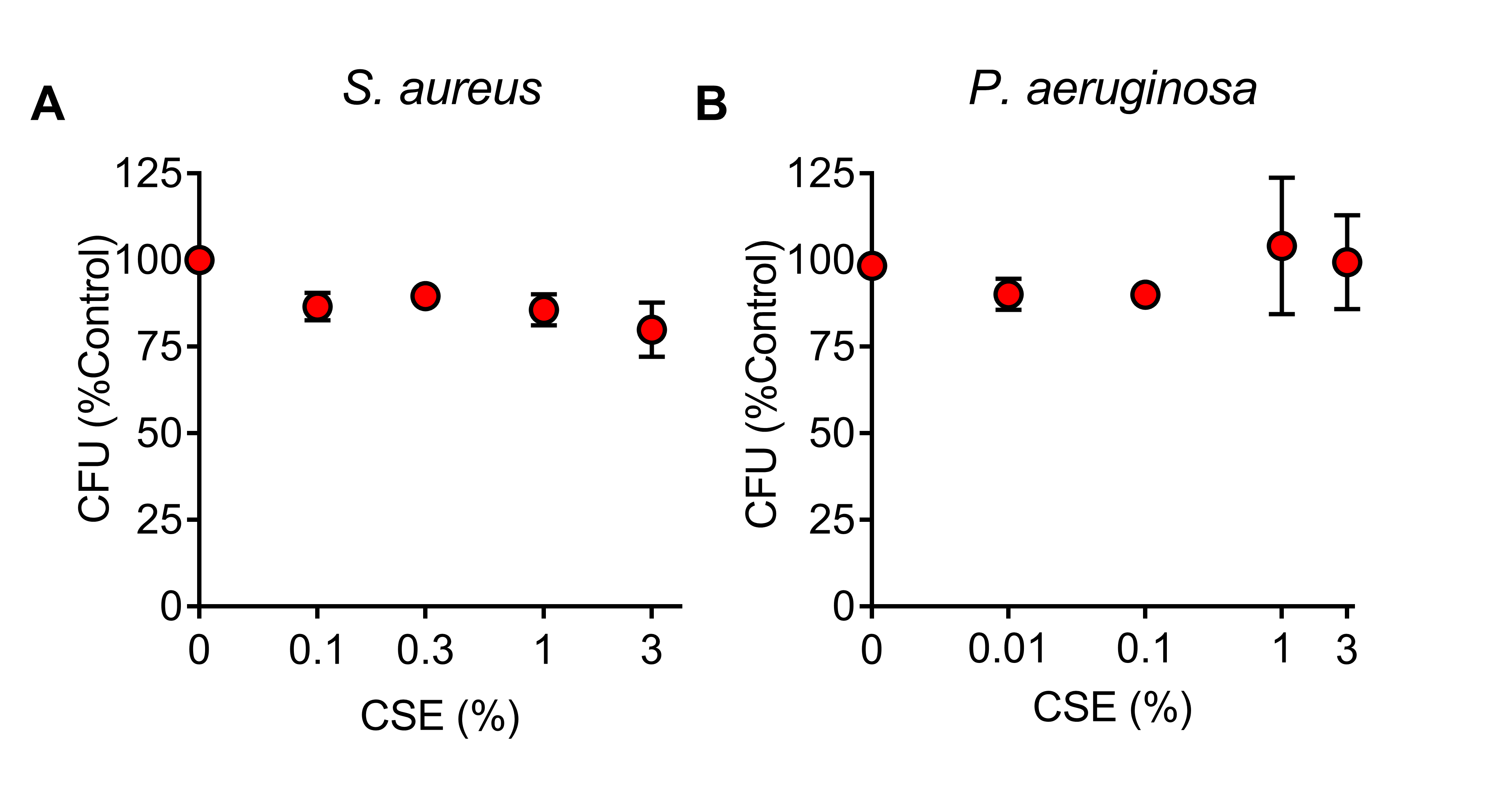

Supplement: Supplementary file 1 — Figure S1. Cigarette smoke extract alone does not increase bacterial growth. (A) S. aureus growth overnight in the presence of increasing concentrations of CSE assessed by CFU. (B) P. aeruginosa overnight growth in the presence of increasing concentrations of CSE assessed by CFU. (TIFF 601 kb) [file 12931_2018_743_MOESM1_ESM.tif]
